# Cancer Survivorship Issues: Dissemination and Translation of Evidence-Based Knowledge

**DOI:** 10.3390/cancers13225794

**Published:** 2021-11-18

**Authors:** Saskia F. A. Duijts, Evelien R. Spelten

**Affiliations:** 1 Research & Development, Netherlands Comprehensive Cancer Organisation (IKNL), Godebaldkwartier 419, 3511 DT Utrecht, The Netherlands; 2 Department of Medical Psychology, Amsterdam University Medical Centers, De Boelelaan 1117, 1081 HV Amsterdam, The Netherlands; 3 Violet Vines Marshman Centre for Rural Health Research, Rural Health School, La Trobe University, Melbourne 3086, Australia; e.spelten@latrobe.edu.au

This issue of Cancers is a Special Issue focusing on ‘cancer survivorship’. Worldwide, there were an estimated 19.3 million new cancer cases in 2020, and it is anticipated that there will be 28.4 million new diagnoses in 2040 [1]. This number continues to increase because of the growth and aging of our population. Survival rates also increase due to improvements in early detection, treatment and supportive care [2]. While many patients experience recurrent periods of disease, develop a second primary cancer or are diagnosed with a less advantageous rare tumor type, currently about half of the patients diagnosed with cancer will survive for 10 years or more [3].

Several definitions of cancer survivorship are available, although none is universally accepted [4]. The concept ‘cancer survivorship’ was first described by Mullan, who identified three phases: acute, extended and permanent survival [5]. Since 2006, the U.S. Institute of Medicine defined cancer survivorship as the entire cancer continuum from initial diagnosis through the remainder of life. Moreover, research and practice often focus on the phase of cancer care that takes place after active cancer treatment, which includes physical, mental and social aspects of living with and after a cancer diagnosis [6].

Through the past decades, much progress has been made towards addressing unmet needs, impact of treatment, models of care and opportunities for health care professionals to enhance survivorship care. However, significant gaps and ample challenges remain. Short- and long-term consequences (e.g., fatigue, cognitive problems, pain), but also late effects (e.g., cardiovascular diseases) can hamper the cancer survivor in daily life. Work ability can be affected, relationships influenced and overall quality of life worsened [7]. As this vulnerable population of cancer survivors is growing, there is not only a need for more public awareness, but also for enhanced expertise and knowledge from (health care) professionals. That is, cancer survivors, with all their issues, will continue to be part of our society, and as a community in oncology, we have a shared responsibility for survivorship care!

In this Special Issue on ‘cancer survivorship’, we have brought together an assembly of original studies and perspectives, focusing on gaps and challenges these patients and (health care) professionals are facing. 

Many facets of cancer survivorship are being considered in a total of 18 manuscripts from 10 different countries. Nine manuscripts presented results for generic populations of cancer patients; three studies focused on patients with colorectal cancer and two on patients with breast cancer. The other studies considered patients with, in random order, esophageal, gynaecological and ovarian cancer, and the final manuscript considered the care provider in the possible new role of community oncologist. The vast majority of studies focused on the patient and patient-related aspects such as fatigue, work, quality of life, sexuality, cognitive symptoms and depression. Around a third of the manuscripts more explicitly addressed care delivery or translated the implications of their work to care delivery.

In their qualitative study, Bennett et al. (2021) explored the experiences of 18 patients who had undergone an esophagectomy, with the aim to gather health education needs of this group. Participants emphasized they did not know what to expect throughout treatment and recovery, which might have led to a traumatic period of adjustment, required because of changes to their physical, psychological and social functioning. Patients mentioned that support provided by family, friends and acquaintances was variable and uninformed, often to the point of being counterproductive to physical and psychosocial recovery. The authors underscore that patients need to be prepared for each stage of their cancer journey, and that families and the wider social networks should receive education that enables them to provide esophageal cancer survivors with appropriate support [8].

Maass et al. (2021), in a cross-sectional study, compared fatigue experienced by long-term breast cancer survivors with that in a reference population, and they evaluated the determinants of this fatigue. The authors found that breast cancer survivors (10 years after diagnosis), more often experienced multidimensional fatigue than the reference group (26.6% versus 15.4%; OR 2.0; 1.4–2.9), and that their fatigue appeared to be associated with symptoms of depression and anxiety. As these symptoms might be modifiable factors that could improve fatigue, if targeted appropriately, general practitioners (GPs) and other (health care) professionals should be vigilant in monitoring these complaints, even long after diagnosis [9].

In a study by de Wind et al. (2021), the risk of unemployment in colorectal cancer survivors was explored. In this nationwide register-based study, persons diagnosed with colorectal cancer (N = 12,007) were compared with a sex- and age-matched population-based reference group (N = 48,028) on loss of paid employment. Colorectal cancer survivors had a higher risk of loss of paid employment (HR 1.56; 1.42–1.71). Within the group of survivors, risk of loss of paid employment was lower for older survivors (>60 vs. 45–55) (HR 0.64; 0.51–0.81) and higher for those with a more advanced cancer stage (IV vs. I) (HR 1.89; 1.33–2.70) and for those receiving radiotherapy (HR 1.37; 1.15–1.63). Based on these findings, the authors advise support for colorectal cancer survivors at high risk of loss of paid employment with work-related interventions as part of cancer survivorship [10].

Gunn et al. (2021) conducted semi-structured interviews with 13 Australian cancer survivors who completed active cancer treatment in an urban center and returned to their rural communities. Additionally, interviews were held with six adults who were caring for a rural/remote cancer survivor, and with three persons who were both survivors and caregivers. An overall theme that emerged from the interviews was the lack of confidence in the ability of rural health services to provide the required help. Explanations included the lack of continuity in the tenure of rural GPs, long waiting times to access services, concerns about quality of care, concerns about the limited scope of medical services provided by rural hospitals, and the lack of availability of quality-of-life-enhancing programs in these settings. To overcome barriers, the authors recommend nurse-led, telephone-based or face-to-face interventions, initiated and continued by the same service provider, including support to manage emotional challenges associated with cancer survivorship [11]. 

A population-based longitudinal study was conducted by Mohlin et al. (2021) to investigate resilience and Health-Related Quality of Life (HRQoL) from diagnosis to 1 year in 418 Swedish women with primary breast cancer (BC). The mean score for resilience in this group was 70.6 (SD = 13.0) at diagnosis and 68.9 (SD = 4.0) after 1 year (*p* < 0.001). Those with a greater level of trust in their treatment and a greater level of satisfaction with the staff–patient encounters, throughout the treatment process, had higher levels of resilience at 1 year post diagnosis. Participants who were more physically active also tended to have higher resilience. Resilience and HRQoL decreased during the first year after diagnosis in the Swedish BC cohort, and the scores were lower than those of the general population at both time points. Importantly, no oncological treatment modality was associated with changes in resilience levels [12].

Kjaer et al. (2021) investigated the risk of depression, and associated factors, in colorectal cancer (CRC) patients. They followed up with 1324 patients with CRC and 6620 matched cancer-free participants for up to 16 years for either a first hospitalization for depression or antidepressant prescription after diagnosis of CRC cancer or study entry date. During follow-up, 191 (14.4%) patients with CRC and 175 (2.6%) cancer-free comparison persons experienced depression. After adjustments, in the first year after cancer diagnosis, patients with CRC had a 12-fold higher hazard compared with the cancer-free population (HR 12.01; 7.89–18.28). The risk decreased during follow-up but remained significantly elevated with an HR of 2.65 (1.61–4.36) after 5 years. Identified risk factors were presence of comorbidities, advanced disease stage and use of radiotherapy, while lifestyle factors and chemotherapy did not seem to contribute to the increased risk. Care professionals should be aware of this late effect of CRC and its treatments, particularly in patients with comorbid conditions, advanced disease stage and patients who are treated with radiotherapy [13].

An online, group-based, videoconferencing-delivered cognitive behavioral therapy (CBT) intervention (‘Recapture Life’) for adolescents and young adults (AYAs) was explored by Sansom-Daly et al. (2021), in a 3-arm randomized controlled trial comparing Recapture Life with an online peer support group and a waitlist control group, with the aim of testing its impact on quality of life, emotional distress and health care service use. There were 40 AYAs within 24 months of completing treatment who participated, together with 18 support persons. No groupwise impacts were measured immediately after the 6-week intervention. However, Recapture Life participants reported using more CBT skills at the 6-week follow-up (OR 5.58; 2.00–15.56, *p* = 0.001) than peer-support controls. Recapture Life participants reported higher perceived negative impact of cancer, anxiety and depression at the 12-month follow-up, compared to peer-support controls. The authors conclude that understanding how best to engage AYAs in psychological support, and at which points in time, remains a challenge [14].

As cancer survivors might experience long-term cognitive symptoms, which can affect their work ability, Klaver et al. (2021) evaluated the trajectories of self-reported cognitive functioning in (partially) work-disabled cancer survivors. In addition, explanatory factors of these trajectories were explored. Participants (N = 206) were assessed on self-reported cognitive functioning at three time points between 2 and 4 years after their first day of sick leave. A statistically significant improvement in cognitive functioning was found in the total group (β = 4.62, SE = 0.91, *p* < 0.001). However, self-perceived cognitive functioning scores remained considerably lower than the mean score of the general population. Cancer survivors who were non-durable work-disabled (partly or fully) reported worse cognitive functioning compared to those assessed as being able to work. Fatigue at 2 years after the first day of sick leave was the only factor found to be associated with cognitive functioning. As cognitive symptoms are a persistent problem in (long-term) work-disabled (partly or fully) cancer survivors, evidence-based treatment options are warranted [15].

Stephenson et al. (2021) considered the challenging finding that cancer patients do not engage with supportive cancer-care programs, despite evidence that these programs enhance physical and psychosocial well-being. Cancer survivors from three countries completed an online questionnaire to investigate the utility of the Common Sense Model of Self-Regulation for predicting supportive-care use. Using this model, some factors appeared to be important in relation to the uptake of supportive care. However, the authors conclude that more clarity is required on the relationship between illness beliefs and coping [16].

With regard to the current perspectives included in this Special Issue of Cancers, Tralongo et al. (2021) make a plea for effective clinical governance of survivorship care to ensure a successful transition between active and post-treatment life, with enhanced quality of life for patients as the primary objective. In their perspective, they focus on the possible role of the so-called ‘community oncologist’. As a trained health professional, and focused on longevity, this professional could represent a management solution in all kinds of intermediate clinical conditions that arise between the hospital specialist, frequently overworked, and the GP, who is often disadvantaged by the lack of specific expertise. The authors state that cooperation between these professionals allows for improvement in health education and professional training, and a more adequate and effective organization of services [17]. 

Next to these more extensively highlighted research papers, numerous other studies on cancer survivorship have been included in this Special Issue, mainly focusing on HRQoL issues in these patients. For example, Chow et al. (2021) examined experiences of Chinese gynaecological cancer patients regarding the effects of treatment on their sexual function and femininity, their relationships and the adequacy of sexual information received from health care professionals. Overall, these patients asked (health care) professionals to proactively initiate discussions on sexual problems [18]. Cortés-Ibañez et al. (2021) used supervised nonlinear algorithms to identify key health behaviors in cancer survivors and to compare the classification performance of linear and nonlinear algorithms when differentiating cancer survivors and cancer-free participants based on health behaviors and socioeconomic factors. No such differentiating health behaviors could be identified in this study [19]. Ton et al. (2021) found that rectal cancer patients are more likely to have sleep complications compared to colon cancer patients. The authors suggest that sleep-focused survivorship care should be adapted according to the CRC site to ensure patients receive appropriate support [20]. Further, Roderburg et al. (2021) evaluated the possibility of an association between cancer and dementia. Findings provide strong evidence for an increased incidence of dementia in a large cohort of patients with different cancer entities. The authors emphasize that awareness of this comorbidity in cancer survivors is paramount [21]. Lisy et al. (2021) examined in a Delphi study ‘what quality criteria do survivorship experts consider to be important in achieving optimal cancer survivorship care?’ [22]. Stegmann et al. (2021) focused in their current perspective on survivorship care questions that can be encountered by patients with prolonged incurable cancer as well [23]. In addition, Dumas et al. (2021) qualitatively explored the lived experience of older patients with advanced ovarian cancer undergoing chemotherapy, their treatment preferences and treatment burden [24]. Finally, Doege et al. (2021) compared HRQoL of survivors of breast, colorectal and prostate cancer (14–24 years post diagnosis) with that of same-aged non-cancer controls. The findings underscored the need for a comprehensive survivorship care program in order to monitor and treat potential late and long-term effects after the diagnosis and treatment of cancer [25].

While developments in cancer survivorship have shown steady and continuous progress over the years, collaborative, (inter)national efforts are still needed to address existing gaps in knowledge and challenges in care. Research findings, both from the work presented in this Special Issue and beyond, need to be translated into practice. It is important that funds and resources are allocated to facilitate this. 

We hope this selection of original studies and perspectives we have presented on cancer survivorship will be useful to both health care professionals and researchers.
